# Utilization and Feasibility of a Wearable Device in Patients With Sedative Effects of Drugs: Protocol for a Prospective Observational Study for the Advanced Respiratory Monitoring Events in Drug Toxicity (ARM-ED) Study

**DOI:** 10.2196/66961

**Published:** 2026-03-19

**Authors:** Lisa Christine Dunlop, Bruce Henderson, Osian Meredith, Chris Trueman, Christopher Carlin, Robert Docking, David J Lowe

**Affiliations:** 1 Emergency Department, Queen Elizabeth University Hospital, 1345 Govan Road, Glasgow, G51 4TF, United Kingdom, 44 0141 452 2930; 2 PneumoWave Ltd, Motherwell, United Kingdom; 3 Department of Respiratory Medicine, Queen Elizabeth University Hospital, Glasgow, United Kingdom; 4 Intensive Care Department, Queen Elizabeth University Hospital, Glasgow, United Kingdom; 5 School of Health and Wellbeing, University of Glasgow, Glasgow, United Kingdom

**Keywords:** wearable device, respiratory biosensor, opioid-induced respiratory depression, illicit drug toxicity, drug toxicity, benzodiazepine toxicity, emergency department

## Abstract

**Background:**

Drug-related deaths worldwide are most commonly attributed to opioids. Opioids and other sedative drugs can cause respiratory depression and airway compromise, leading to hypoxia and death. Device technology and artificial intelligence used to detect drug overdose has the potential to improve outcomes. PneumoWave Ltd has developed a small chest-worn respiratory monitoring device to detect concerning breathing patterns and alert an emergency response.

**Objective:**

The aim of this study is to investigate the feasibility of using the PneumoWave device in hospital patients at risk of respiratory depression.

**Methods:**

This 18-month prospective observational study was performed at the Queen Elizabeth University Hospital, Glasgow. The study investigates the use of the device on 3 groups of patients at risk of respiratory depression due to drugs. This includes patients attending the emergency department (ED) due to sedative drug overdose, patients receiving procedural sedation and analgesia in the ED, and patients receiving general anesthesia in theaters. Consenting participants will have the PneumoWave sensor paired with end-tidal CO_2_ monitoring and regular recordings of vital signs. Usability will be tested by administering a questionnaire to the patient, the clinician, and the nurse. The primary end point is to determine the feasibility of gathering respiratory data from a wearable respiratory monitoring device in the ED. Statistical analysis includes comparison of biosensor data against reference physiology time course data.

**Results:**

Trial recruitment was completed on December 8, 2023. Twenty-five patients were enrolled in group 1 (acute toxicity); 39 patients in group 2 (procedural sedation), including 1 patient who was subsequently withdrawn; and 14 patients in group 3 (acute toxicity), with 2 patients withdrawn. Publication of the study results is anticipated by December 2025.

**Conclusions:**

This study will explore the feasibility of the PneumoWave device in a variety of clinical contexts in which risk of pharmacologically induced respiratory depression is present. This will provide valuable insight into the use of device technology in individuals at risk of illicit drug–related harm within the relative safety of a hospital setting. A limitation of the study procedure is exclusion of patients with intoxication after sedative drug overdose who lack the capacity to provide informed consent. This study has been designed to acquire feasibility data to demonstrate the potential for continuous respiratory monitoring to improve outcomes for patients who are at risk of drug-induced respiratory depression, inform product development, and inform the design of future pivotal clinical investigations.

## Introduction

### Background

Over a quarter of a billion people worldwide used illicit drugs at least once during the year 2021, 60 million of which used opioids. Overdose is a significant cause of preventable morbidity and mortality globally, with approximately 128,000 deaths directly due to drug use in 2019 [1]. Within Europe, Scotland reports the highest drug-related death rate at 19.8 per 100,000 population. This study is sited in a tertiary urban hospital in Glasgow, Scotland. National Health Service (NHS) Greater Glasgow and Clyde Health Board has the highest drug-related death rate in Scotland (34 per 100,000 in 2022), and hence, this research is at the epicenter of the drug death crisis [2].

Opioids are the drug group most commonly attributed to drug-related death in the world [3]. In 2021, opioids were implicated in 75% and 74% of drug-related deaths in the United States and Europe, respectively [4,5]. Similarly, in Scotland, postmortem toxicology reports found that the most commonly implicated drugs were opioids (n=1051, 82.5%) and benzodiazepines (n=1051, 57.2%) [2].

Opioids are μ-receptor agonists, and their overdose can lead to reduced consciousness, irregular or slow breathing patterns, and potentially life-threatening or fatal respiratory depression. Death following the use of opioids is due to loss of respiratory reflexes, apnea, and respiratory failure [6,7]. There is an increased risk of death in opiate-naïve patients when opioids are combined with other central nervous system (CNS) depressant drugs such as alcohol, benzodiazepines, and other co-prescribed medications such as gabapentinoids [8,9]. Overdose of benzodiazepines, like opioids, can lead to reduced consciousness and loss of airway reflexes, leading to potential death. This is the case especially when taken in the context of user naivety, high doses used, potent preparations, or co-ingestion [10,11].

Access to emergency services and the use of the opioid antagonist, naloxone, given by any individual can reduce the risk following drug overdose [12]. A user who experiences an overdose must, however, be able to access help and therefore is at higher risk of death when using drugs alone [13].

The use of technology may help provide a solution to reduce the risk of fatal overdose in drug users. There have been several studies investigating the use of device technology in this setting, and these devices may be attached to or positioned somewhere away from the individual. Mobile phone apps are available for prevention and detection of harm. These often rely on the user (who may be unconscious) being able to alert help via the device [14]. A mobile app described by Nandakumar et al [15] found that a mobile phone which was adapted to use sonar to detect respiratory effort and apnea reported higher performance in the detection of postopioid self-administration central apneic events (10 s or longer). However, the device was required to be held by a second person at a 90° angle and at a distance of just 1 m from the individual for its successful use. Other near-patient devices include in-room movement sensors and help buttons; the latter again require the user to be capable of pressing the button [16,17].

Several wearable sensors in development have been described, including those that deploy naloxone [18-20]. The described devices have major barriers to their potential use. Mesa et al [19] describe a sensor that requires implantation of a drug-delivery system, which would likely not be acceptable to many individuals, with a potential increased risk of infection. Chan et al [20] describe a bulky device, which may reduce compliance of use, and they found it was unreliable in deploying the naloxone. Roth et al [21] describe a commercially available biosensor to detect movement. They showed success in collecting accelerometer data; however, they detected no respiratory depression despite 385 drug administrations recorded (>90% involved fentanyl), 27 of which the user described themselves as having “too much”.

There are some potential barriers to the use of a device such as battery life, effective functioning, size, weight, and aesthetics [22,23]. Despite this, potential users of devices are likely to be accepting the device if they understand its ability to reduce harm [24]. As described, the near-patient device described by Nandakumar et al was limited by its requirement to be perpendicular and within 1 m proximity to the patient to achieve stated results [15]. There is clearly a need for innovative technology that can detect individuals at risk of overdose, enabling help to reach them, while also being acceptable to wear.

### Wearable Technology

PneumoWave Ltd has developed a small, wearable biosensor that incorporates a microelectromechanical system inertial motion unit capable of continuously monitoring central body motion over time [25]. This physical monitor is linked wirelessly (Bluetooth) to central monitoring systems via either a data gateway that plugs into a nearby electrical socket or a smartphone. The company’s technology is separated into two product lines: DC (data collection) and the Alert (event recognition).

**Figure 1.**
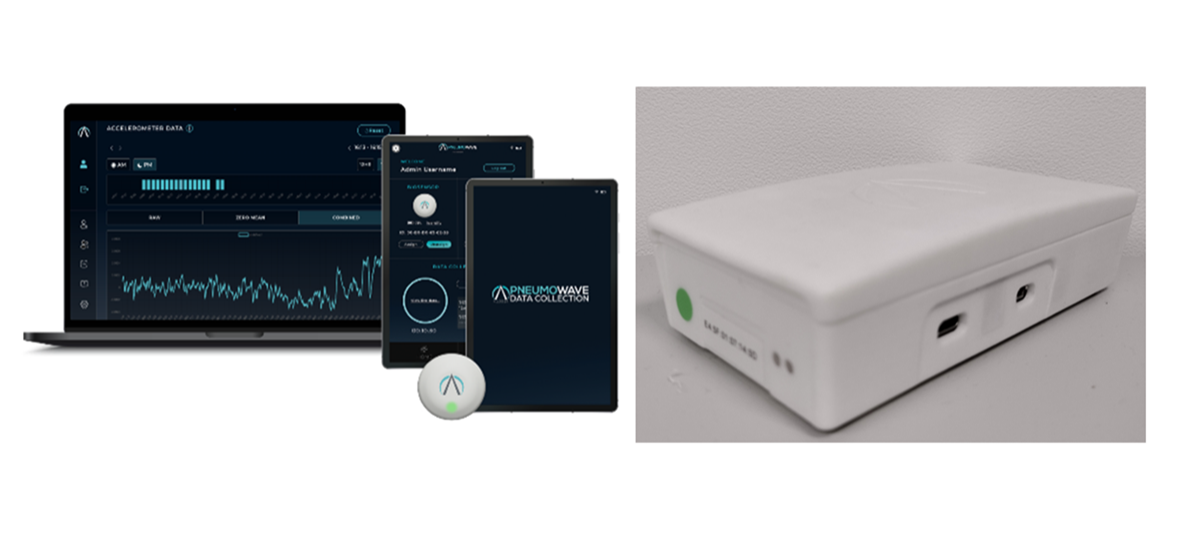
PneumoWave data collection system. A clinical data collection and analysis platform showing the biosensor, mobile app, or hub (right image), and clinician web portal with visualization of motion data. This system is designed for researchers and clinicians, with a focus on comprehensive data collection and retrospective analysis.

PneumoWave DC is a UKCA (UK Conformity Assessed) Class 1 medical device with the following intended use: PneumoWave DC is a monitoring device intended to capture and store chest motion data continuously for retrospective analysis by a health care professional to document physical movement associated with applications in physiological monitoring. The device is not intended for diagnosis of respiratory conditions. It is indicated for patients deemed to be suitable for chest motion monitoring in health care facilities by a physician (Figure 1).

Prototype biosensor units (identical mounting methods and similar dimensions) were subject to two previous studies resulting in 16,476 hours of wear in 138 participants, indicating a high tolerance for the design (NCT04668313 and ISRCTN12060022). The DCM system collects raw X, Y, and Z axis accelerometer movement data with no data processing applied, which transmits via the App or Hub to a cloud environment. By accessing the cloud dashboard, users are able to view and download the accelerometer data.

**Figure 2.**
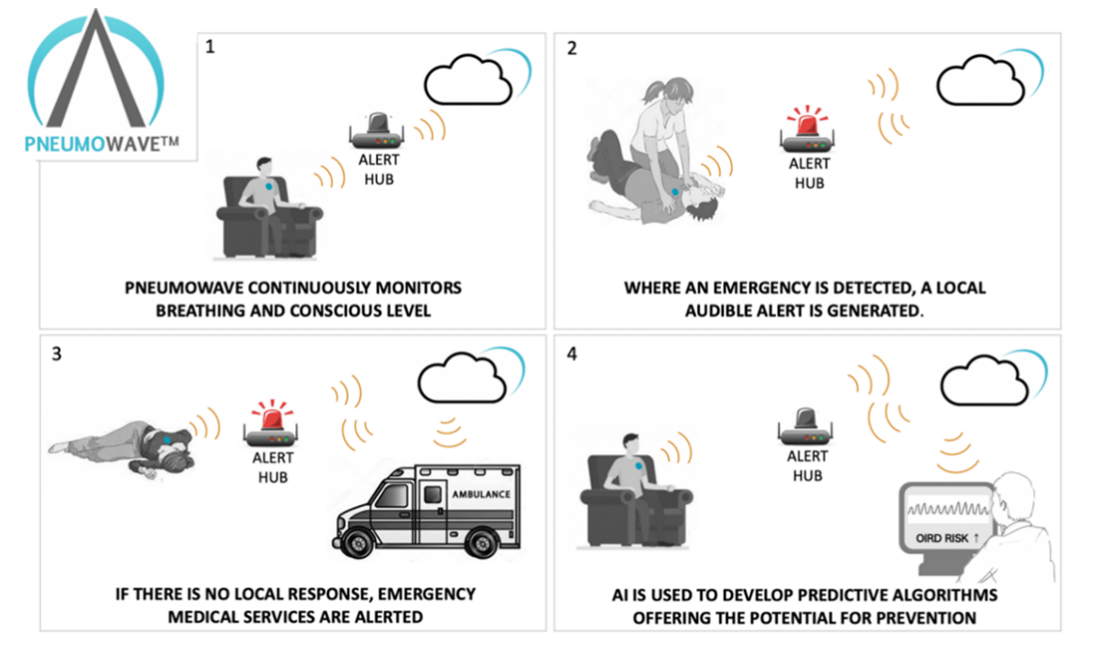
PneumoWave ALERT platform technology overview. The biosensor will detect concerning breathing patterns and will send an alert for urgent help for the individual following overdose.

The ALERT system for real-time collection of data plus processing is in development to enable detection and the potential of immediate intervention through algorithms currently under development and is a key driver for this study (see Figure 2).

This research investigates the feasibility of the use of the PneumoWave Ltd wearable biosensor in individuals at risk of drug-induced respiratory depression. The safety of a hospital setting has been utilized to understand the feasibility of use. Risk of respiratory depression occurs in a variety of situations within hospitals. As described, an overdose of opioids or other sedating/respiratory depressant drugs often requires medical care and attending the emergency department (ED) for treatment [26]. This group of patients is included in this trial and provides a pragmatic indication of feasibility of use in patients at risk of respiratory depression. Opioids and sedatives are also administered in hospital for medical purposes in a setting where the patient will need respiratory monitoring. One such example is procedural sedation and analgesia (PSA). This is a technique in which sedation is given with an aim to allow a painful procedure to be performed but not deep enough to cause apnea, though there is a risk, and this occasionally does occur [27]. A further example is that of induction for a general anesthetic (GA) where apnea is either certain (with muscle relaxant) or likely (during GA induction). These two groups of patients will also be studied to understand the use of the device in settings of increased risk or definite apnea due to drug administration. End-tidal CO_2_ (ETCO_2_) is used in hospitals in these settings to monitor respiratory rate by capnography and as such is the gold standard comparator in this study [28].

### Study Hypothesis

The PneumoWave device will be well tolerated by patients and will successfully capture respiratory effort and patient movement accelerometer data.

### Objectives

The primary objective of this study was to assess the feasibility of collecting respiratory waveform data using the PneumoWave device in ED patients from groups 1‐3 (see Study Population).

Secondary objectives of the study were as follows: (1) Collate respiratory data from the PneumoWave device and compare to ETCO_2_ in patients from all 3 groups. Respiratory data will observe normal respiratory effort, reduced respiratory effort due to sedating drugs or anesthetic agents, and response to treatments and interventions required in patients with reduced respiratory effort. (2) Collate waveform and motion artifact data from the PneumoWave device in patients from all 3 groups to observe the relationship with the individual’s conscious level. (3) Assess the usability of PneumoWave in the opinion of the clinician, nurse, and patient. (4) Understand the feasibility of gathering respiratory data from a wearable respiratory monitoring device in the ED.

## Methods

### Study Design

This is a single-center observational cohort study conducted using passive non-invasive data collection.

### Setting

This study was undertaken at the Queen Elizabeth University Hospital, Glasgow, United Kingdom, over an 18-month period from June 9, 2022, to December 8, 2023.

### Study Population

The overall study population consists of patients at risk of respiratory depression due to exposure to sedating drugs. The study observes three distinct patient groups:

- Group 1 (acute toxicity group): Patients who present to the ED with actual or potential CNS or respiratory depression secondary to toxicological cause
- Group 2 (PSA group): Patients who undergo PSA with respiratory and CNS depressant drugs in the ED
- Group 3 (GA group): Patients who undergo GA in the anesthetic room in theater

### Inclusion Criteria

The inclusion criteria are presented in Table 1.

**Table 1.** List of inclusion criteria for the Advanced Respiratory Monitoring in the Emergency Department (ARM-ED) study.

Group	Inclusion criteria
All groups	Age >16 years; able and willing (in the investigators’ opinion) to comply with all study requirements; able to speak and read English
Group 1 (acute toxicity)	Willing and able to give informed consent (or via Welfare Attorney, Guardian, or Nearest Relative); presenting to ED^a^ due to presumed overdose of a drug with potential for respiratory depression (intentional, accidental, recreational, or therapeutic excess); having at least one of the following: Glasgow Coma Score (GCS) <15, respiratory depression, or risk of deterioration in GCS or respiration
Group 2 (PSA^b^)	Willing and able to give informed consent; undergoing procedural sedation and anesthesia in ED
Group 3 (GA^c^)	Willing and able to give informed consent; undergoing elective general anesthetic of any type in the anesthetic room

a ED: emergency department.

b PSA: procedural sedation and analgesia.

c GA: general anesthetic.

### Exclusion Criteria

The exclusion criteria are presented in Textbox 1.

Textbox 1. **List of exclusion criteria for the Advanced Respiratory Monitoring in the Emergency Department (ARM-ED) study.**

- Unable to provide consent and no next of kin to provide consent on participant’s behalf
- Impaired consciousness or respiratory suppression most likely due to cause other than acute drug use
- Condition primarily related to alcohol use and no evidence of acute drug use
- Condition due to withdrawal of drugs or alcohol
- Treating clinician deems patient inappropriate to be included in the study
- Pregnant female
- Patient has implantable device (eg, pacemaker)

### Withdrawal of Participants

Participants will be able to withdraw from the study at any time, without impact on their clinical management.

### Sample Size and Rationale

This is a feasibility study to generate data for future prospective interventional studies; therefore, no formal sample size will be generated. The research group planned a target of 50 patients recruited to both groups 1 and 2 as the number of minutes of data capture would successfully answer the primary outcome question of feasibility. Group 3 was included following trial commencement. Following evidence of the volume and type of data being produced from groups 1 and 2, a target of 12 was used based on the understanding of the number of minutes of data gathered. We aim to recruit a total of 112 patients to the study, which will provide adequate data to establish feasibility of use for larger studies. The sample size breakdown is as follows: group 1: 50 patients; group 2: 50 patients; and group 3: 12 patients.

### Primary End Points

The primary end point is understanding the feasibility of gathering respiratory data from a wearable respiratory monitoring device in the ED.

### Secondary End Points

The secondary end points are as follows:

- Add to existing respiratory data to inform machine learning and improve sensitivity and specificity of PneumoWave device
- Understand the usability of the device within acute care setting
- Understand the feasibility of gathering respiratory data from a wearable respiratory monitoring device in patients undergoing GA

### Outcome Measures

The primary outcome measures will measure the length of time the device is in situ on the patient, the number of times the device is removed by the patient or others, and the ability of the device to collect data while in situ. The secondary outcome measures will observe waveform data from the PneumoWave device and compare those with normal care vital signs and continuous monitoring vital signs extracted from patient monitors. PneumoWave respiratory wave patterns and motion artifact will be compared to clinical events, including normal respiratory patterns, clinical deterioration, and interventions in ED or in anesthetic room in theater, and Glasgow Coma Score.

Stakeholders will be interviewed regarding the usability of the device. These stakeholders are the clinician and nurse caring for the participating patient in question, and the patient themselves, if able to.

Outcome measures and their associated data sources and data capture are shown in Table 2.

**Table 2.** Outcome measures and associated data source and data to be collected.

Outcome measure	Data source	Data to be collected
Primary outcome measures		
Length of time the device is in situ on patient	CASTOR EDC [29]	The total time device present on chest as documented by researcher (seconds)
The number of times the device is removed by the patient or others	CASTOR EDC	Absolute number of times the device was removed
The ability of the device to collect data while in situ	PneumoWave sensor	Total amount data recorded (seconds) as the proportion of time the device was on chest (seconds)
Secondary outcome measures		
PneumoWave device waveform data comparison to clinical events	PneumoWave device and CASTOR EDC	Visual representation of PneumoWave data and comparison to the following: ETCO_2_^a^ waveforms Documented respiratory rate Interventions GCS^b^
Usability of device	CASTOR EDC	Data from results of interview reported in Likert scale from 1 to 5.

a ETCO_2_: end-tidal carbon dioxide.

b GCS: Glasgow Coma Score.

### Study Procedures

#### Potential Participant Identification

##### Group 1 (Acute Toxicity) and Group 2 (PSA) Patient Identification:

Patients from group 1 and group 2 will be recruited concurrently and opportunistically. Patients will be recruited during the research team by the researcher who will observe ED attendances remotely via electronic health records and identify potential participants who are either intoxicated (group 1) or may undergo procedural sedation (group 2). They will assess the patient for eligibility. ED staff will be given information on study recruitment and alert the research team to assess eligibility for inclusion.

##### Group 3 (GA) Patient Identification

Patients will be recruited during specified elective theater lists undergoing a GA. Patients will be approached by the anesthetist for willingness to participate, with the researcher assessing for eligibility, providing them with the patient information sheet, and gaining consent from the patient.

### Consent

Written consent will be gained from the patient or Welfare Attorney/Welfare Guardian/Nearest Relative for group 1 and from the patient only for group 2 and group 3.

### Source Data Collection and Patient Observation

Source data will be collated from multiple sources, including study participant interviews; usual care electronic health records; anesthetic charts; medical, ambulance, and nursing notes; drug charts; and observation charts. Continuous vital sign data will be downloaded from the standard ED or anesthetic monitors or study monitors, PneumoWave sensor data, and manual respiratory rate for study periods using ETCO_2_ monitor.

During the study episode, data will be collected from the above sources for a period of time specific to each group. Data are all gathered by the researcher. Group 1 patients will be observed by the research team member for 1 hour or from commencement of data collection until they are discharged or moved from the ED, whichever is shorter. Group 2 participants will be observed for the duration of their procedural sedation, length of which may vary, and recovery period for up to 15 minutes. Data are gathered from group 3 participants prior to their GA while in the theater’s anesthetic room, during induction of anesthesia and until the patient is moved to the theater.

Usability is investigated by interviewing stakeholders (patient, nurse, and clinician) following each respective research study episode. It is expected that due to the nature of the study, it may not be possible to gather these data for all patients recruited due to reduced conscious level. Responses to domains were on a scale of 1 (least positive) to 5 (most positive). The domains that were investigated were perceptions on device comfort, device intrusiveness, device safety, delay to treatment, acceptability of device in normal practice.

### Review of Notes at 28 Days

The participant has a follow-up review of clinical status at a single time point of 28 days postrecruitment via electronic case records. This will identify any adverse outcome relating to their ED attendance or anesthetic, for example, need for critical care, disability, morbidity, or mortality as well as identify any relevant usual care drug screens performed at the time of assessment.

### Waveform Analysis and Predictive Modeling Algorithms

Data from PneumoWave are analyzed in parallel with deidentified clinical and vital sign observational data to build artificial intelligence–driven predictive modeling algorithms.

### Summary Study Procedure

Table 3 shows a summary of study procedures, including participant enrollment, interventions, and assessments (Checklist 1).

**Table 3.** Standard Protocol Items: Recommendations for Interventional Trials (SPIRIT) figure of enrollment, interventions, and assessments.

Study procedure	Screening/consent (first visit)	Research episode	28 days’ follow-up electronically
Obtain informed consent where relevant	✓		
Review inclusion/exclusion criteria	✓		
Complete case report form	✓	✓	✓
Collect continuous data from standard care vital sign monitor		✓	
Collect waveform respiratory data		✓	
Review retrospective notes for adverse outcome/available toxicology screen			✓

### Data Collection and Management

CASTOR (The Netherlands) [29] will provide a platform for data collection via an electronic case report form [29]. CASTOR EDC is compliant with 21 CFR Part 11, ICH E6 GCP, GDPR, and HIPAA. It is ISO27001 and ISO9001 certified.

### Safety and Adverse Events

Adverse events and serious adverse events (SAEs) will be managed in line with Good Clinical Practice guidelines. SAEs meeting the above criteria must be reported to the Pharmacovigilance Office immediately (within 24 h) using the SAE form for a non-CTIMP. All SAEs will be reported to the ethics committee within 15 days of the chief investigator or delegate becoming aware of the event.

### Planned Analysis

#### Primary Outcome Measure Analysis

Descriptive statistics will be used to analyze the primary outcome measures and assess the feasibility of studying the use of the PneumoWave device in the three patient groups. The analysis for each primary outcome measure is as shown below.

- Length of time the device is in situ on the patient: The time between the documented time the device is placed on the patient and time off the patient will be calculated (seconds). Any time the device was removed will be deducted from the total time. A final analysis of median, mean, and associated IQR and SD will be reported for each group.
- Number of times the device was removed by the patient or others: This will be reported as median, mean, and associated IQR and SD, respectively, for each group.
- Ability of the device to collect data while in situ: The amount of data gathered for each episode will be recorded and compared to the length of time the device is in situ on the patient’s body. This will be reported as a proportion. The median, mean, and associated IQR and SD will be reported for each group.

#### Secondary Outcome Measure Analysis

The secondary outcome measures study the data that is captured by the PneumoWave device. In order to analyze this, the device data will be visually reviewed alongside the usual care vital signs and continuous monitoring signs. Respiratory rate calculated from the PneumoWave monitor will be compared to respiratory rate counted by a medical professional from the ETCO_2_ monitor. Accuracy will be analyzed through sensitivity and specificity tests using ETCO_2_ measurements as the gold standard.

Data points in which there has been a change in respiratory pattern, clinical deterioration, clinical intervention, and Glasgow Coma Score will be visually reviewed through time stamp looking at both usual care vital signs and PneumoWave study waveform data. Results will be presented both through written description and visually to highlight waveform changes if applicable.

Analysis of clinician, nurse, and patient opinions on the use of the device will be recorded as median and ranges.

Feasibility was defined as the device remaining attached to the chest with two or fewer removals during the monitoring period and achieving data capture for more than 75% of the total wear time.

The statistical analysis will be performed using MiniTab.

### Ethical Considerations

#### Ethics Approval and Consent to Participate

Favorable ethical approval was obtained from Scotland A Research Ethics Committee (REC) prior to study commencement on January 19, 2022 (REC reference: 21/SS/0083, Integrated Research Application System project ID: 303922).

A substantial amendment was submitted on March 28, 2023, and gained favorable opinion on April 28, 2023, by Scotland A REC.

The trial was registered on March 30, 2022, on ClinicalTrials.gov (reference: NCT05358132). The protocol information presented here has current ethical approval and is from protocol version 2.0.

Any protocol modifications will be approved with the REC prior to initiation of the change. Consent was obtained according to the protocol from patients or a nominated person by the co-investigators of the trial or a suitably trained member of research staff.

#### Process for Informed Consent

The process for informed consent will differ between group 1: acute toxicity group and group 2: PSA group. Group 3: GA group will follow the same consenting process as Group 2.

##### Group 1: Acute Toxicity Group

Because the nature of the condition is being studied, this patient group will most likely be incapacitated and will not be able to provide informed consent. We anticipate that this may lead to difficulty in recruiting patients for this group.

In some cases, the potential study participant will have the capacity either briefly or indefinitely. During this period, the researcher will assess the capacity and will gain informed consent from the participant. The participant will be approached with a verbal explanation from the researcher and provided with a patient information sheet (PIS) to read. They will be given as much time as required to consider the information. When the participant is satisfied and any questions are answered, they will be asked if they will consent to the study. The participant will sign and date the most recent approved version of the informed consent form before any study-specific procedures are performed. The participant will be given a copy of the signed consent form as well as the PIS.

If the patient is unlikely to regain capacity, then as per the Adults with Incapacity (Scotland) Act 2000, consent must be gained from guardian or welfare attorney or nearest relative. The next of kin of the potential participant will be contacted, and verbal and written information regarding the study will be provided. They will be given as much time as required, and a researcher will be available to answer any questions. The next of kin will be required to provide written informed consent and as such must be present in the ED for this.

##### Group 2 (PSA Group) and Group 3 (GA Group)

These patient groups will only be included if they have the capacity to provide informed consent for this study. The participants will be approached with verbal explanation from the researcher and provided with a PIS to read. They will be given as much time as required to consider the information. When the participant is satisfied and any questions are answered, they will be asked if they will consent to the study. The participant will sign and date the most recent approved version of the informed consent form before any study-specific procedures are performed. The participant will be given a copy of the signed consent form as well as the PIS.

In groups 1, 2, and 3, the participant and their Welfare Attorney, Welfare Guardian, or Nearest Relative are free to withdraw from the study at any time for any reason without prejudice to future care and with no obligation to give the reason for withdrawal.

Consent for publication was achieved during initial participant consent.

### Privacy and Confidentiality

Data are anonymized during this study. At study completion, the comprehensive anonymized study dataset will be submitted for inclusion in NHS Greater Glasgow and Clyde SafeHaven. This will allow subsequent deidentified review of all study outcomes, reanalysis of the dataset, and contribution of the dataset to future research work within NHS Scotland.

### Compensation Details

No financial or material compensation was provided to participants for their participation in this study.

### Updates to Protocol Since Registry

There were no significant protocol deviations during the study period. There were several amendments to the protocol following original ethical approval and trial registry. These amendments were reported and approved through the Integrated Research Application System. Table 4 outlines the amendments to the protocol.

**Table 4.** Study amendments following original ethics approval and trial registry.

Sponsor amendment date	Amendment number	Amendment details
March 17, 2022	NSA01	The electronic case report form platform has been changed from Galen to CASTOR EDC [29]. This was changed due to access to the platform. CASTOR is compliant with 21 Code of Federal Regulations Part 11, International Council for Harmonisation of Technical Requirements for Pharmaceuticals for Human Use E6 GCP^a^, General Data Protection Regulation, and Health Insurance Portability and Accountability Act. ISO (International Organization for Standardization) 27001 and ISO 9001 certified. Following research group consensus, the name of the study was changed from TARS (Toxicology Advanced Respiratory Study) to ARM-ED (Advanced Respiratory Monitoring in the Emergency Department). In the protocol, the wording was changed from “participants will be studied for the duration of their ED^b^ attendance” to “participants will be studied for a period of time during their ED attendance.” The methods were updated to include the requirement of a clinician to count the respiratory rate retrospectively. They would do this by reviewing the end-tidal CO_2_ monitor data. The reason for this is to improve the accuracy of respiratory rate counting in the case that the vital signs monitor may not accurately do this.
April 1, 2022	NSA02	A minor amendment was made to exclude pregnant individuals in groups 1 and 2. The reason for the exclusion is that the physiology of breathing is different during pregnancy, and so the respiratory device may not be reliable in this instance. Separate studies are required to investigate the use of the device on pregnant females. In addition, in the emergency department, pregnant females are very rarely sedated for procedures.
June 7, 2022	NSA03	The PneumoWave Biosensor was approved as UKCA (UK Conformity Assessed) Class 1. The documentation states that the sensor will be class 2. This was changed in the protocol.
July 21, 2022	NSA04	Patient information sheets and corresponding consent forms were updated to reflect the correct version date.
March 28, 2023	SA 05	A major amendment was approved to add a third arm to the study, named group 3: general anesthetic (GA) substudy. This group aimed to have a recruitment target of 12 patients to investigate the feasibility of the use of the PneumoWave device in patients who are undergoing an elective general anesthetic in the hospital operating theater anesthetic room. The reason that this additional arm was added was to capture respiratory movement waveform data in patients who would likely have a period of apnea during the anesthetic. The protocol, standard operating procedure, and electronic case report form guidance were updated to exclude patients with an implantable device as per the PneumoWave device instructions for use.

a GCP: Good Clinical Practice.

b ED: emergency department.

## Results

Trial recruitment was completed on December 8, 2023. A total of 25 patients were enrolled in group 1 (acute toxicity), 39 patients in group 2 (procedural sedation), including 1 patient who was subsequently withdrawn, and 14 patients in group 3 (acute toxicity), with 2 patients withdrawn. All withdrawn participants were excluded at an early stage, as planned sedation was not carried out.

Data analysis is currently in progress, and publication of the study results is anticipated by December 2025.

## Discussion

### Expected Principal Findings

The anticipated main finding of this feasibility study is that the PneumoWave wearable respiratory monitoring device will successfully gather respiratory waveform data and will be well tolerated by patients at risk of drug-related respiratory depression. Specifically, it is expected that the device will provide high-quality respiratory waveform data comparable to the gold standard of ETCO_2_ monitoring within the relative safety of the hospital environment [27]. These findings would support the feasibility of using wearable respiratory monitoring to enhance clinical surveillance in populations at risk of respiratory compromise.

The study will allow understanding of the ability of the device to measure respiratory rate and detect changes to a patient’s condition accurately. Apnea can be difficult to study, and in hospitals, it is often brief, as intervention is swift while in the care of medical professionals due to substantial risk of harm if left untreated. Therefore, insight into the feasibility of studying this will be invaluable.

### Comparison to Prior Work

Wearable respiratory monitoring technologies have shown promise in both hospital and community settings, yet are often not acceptable or practical for the patient [15,18-20]. This study, therefore, extends the current literature by focusing on the feasibility of a novel wearable solution that offers continuous and noninvasive respiratory monitoring of the individual at risk of drug-induced respiratory depression.

### Strengths and Limitations

A key strength of this feasibility study is its pragmatic design, enabling evaluation of the PneumoWave device in the ED or anesthetic room, where risk to the patient can be minimized. However, several limitations should be acknowledged. Recruitment challenges are anticipated due to legal restrictions under Scottish Adults with Incapacity laws, which preclude consent via a professional legal representative; this constraint may particularly impact enrollment in the acute toxicology cohort (Group 1). This study has, however, been designed to improve patient outcomes who are at risk of drug-induced respiratory depression and inform future development of clinical support and prediction capability. The study will not be powered to assess diagnostic accuracy or clinical outcomes, and conclusions will therefore be limited to feasibility, tolerability, and data acquisition parameters.

### Future Directions

Findings from this feasibility study will inform the design of larger scale observational and randomized diagnostic accuracy trials in both hospital and supervised injecting settings. Future studies could expand to assess real-time clinical decision support, predictive analytics for respiratory compromise. Long-term goals include the development of predictive models integrating wearable-derived respiratory data with other physiological or behavioral indicators of overdose risk.

### Dissemination

Upon completion of the study, findings will be disseminated through peer-review journal publication and presentation at conferences and engagement with both clinical and public health stakeholders to reduce harm caused by drug-induced respiratory depression.

### Conclusion

This hospital-based respiratory monitoring study will provide a safe environment to understand the feasibility of use of a wearable device in detecting drug-induced respiratory depression and inform measures in reducing associated harm.

## Supplementary material

10.2196/66961 Checklist 1 SPIRIT checklist.

## Abbreviations

ARM-ED: Advanced Respiratory Monitoring in the Emergency Department
CNS: central nervous system
ED: emergency department
ETCO2: end-tidal CO2
GA: general anesthetic
NHS: National Health Service
PIS: patient information sheet
PSA: procedural sedation and analgesia
REC: Research Ethics Committee
SAE: serious adverse event
